# The Lightweight Autonomous Vehicle Self-Diagnosis (LAVS) Using Machine Learning Based on Sensors and Multi-Protocol IoT Gateway

**DOI:** 10.3390/s19112534

**Published:** 2019-06-03

**Authors:** YiNa Jeong, SuRak Son, ByungKwan Lee

**Affiliations:** Department of Computer Engineering, Catholic Kwandong University, Gangneung 25601, Korea; lupinus07@nate.com (Y.J.); sonsur@naver.com (S.S.)

**Keywords:** in vehicle-diagnosis, random forest, IoV, integrated gateway, neural network

## Abstract

This paper proposes the lightweight autonomous vehicle self-diagnosis (LAVS) using machine learning based on sensors and the internet of things (IoT) gateway. It collects sensor data from in-vehicle sensors and changes the sensor data to sensor messages as it passes through protocol buses. The changed messages are divided into header information, sensor messages, and payloads and they are stored in an address table, a message queue, and a data collection table separately. In sequence, the sensor messages are converted to the message type of the other protocol and the payloads are transferred to an in-vehicle diagnosis module (In-VDM). The LAVS informs the diagnosis result of Cloud or road side unit(RSU) by the internet of vehicles (IoV) and of drivers by Bluetooth. To design the LAVS, the following two modules are needed. First, a multi-protocol integrated gateway module (MIGM) converts sensor messages for communication between two different protocols, transfers the extracted payloads to the In-VDM, and performs IoV to transfer the diagnosis result and payloads to the Cloud through wireless access in vehicular environment(WAVE). Second, the In-VDM uses random forest to diagnose parts of the vehicle, and delivers the results of the random forest as an input to the neural network to diagnose the total condition of the vehicle. Since the In-VDM uses them for self-diagnosis, it can diagnose a vehicle with efficiency. In addition, because the LAVS converts payloads to a WAVE message and uses IoV to transfer the WAVE messages to RSU or the Cloud, it prevents accidents in advance by informing the vehicle condition of drivers rapidly.

## 1. Introduction

Due to the introduction of the Fourth Industrial Revolution, information and communication technology (ICT) technology has been applied in various industries and is being developed. Among them, the automobile industry is actively utilizing internet of things (IoT) technology for autonomous vehicles. In addition, many automotive companies around the world are busy developing their technology through various competitions and interactions.

Autonomous driving technology is divided into 0–5 levels. Level 0 means no autonomous driving. Level 1 is a state where one autonomous driving technology is applied. In level 2, an advanced driver assistance system (ADAS) on the vehicle can itself actually control both steering and braking/accelerating simultaneously under some circumstances. The human driver must continue to pay full attention at all times and perform the rest of the driving tasks. Level 3 is incomplete but is capable of autonomous driving and requires the driver to be ready to drive in an emergency. Level 4 is the stage in which the automatic driving system (ADS) of a vehicle performs all the driving tasks and monitors the driving environment in specific situations. The driver does not need to pay attention in such situations. Level 5 is a fully autonomous car that does not require a person to drive at all [1]. In the automobile industry, development has been achieved satisfying level 4, but commercialization has not yet been achieved. In order to commercialize autonomous unmanned vehicles, there are several problems to be solved such as the stabilization of software and the perception change about safety.

The first problem to be solved is to improve a communication environment. The autonomous vehicle is equipped with a variety of internal sensors to collect data, and the collected data is exchanged with the Cloud server using a vehicle communication protocol. At this time, the Cloud server combines the data received from the sensor with various algorithm formulas to organically transmit commands to the vehicle or generate new data. There are various sensors in the vehicle to collect data. As the number of sensors gets increased, a vehicle uses several kinds of protocols for communication. Existing vehicle gateways, however, did not support some of the major protocols used in the vehicle, or rapid communication.

The second problem is to extend the scope of vehicle diagnosis. Autonomous vehicles perform their own diagnosis and driving simultaneously. Since existing vehicles use on board diagnostics (OBD) to perform fault-diagnosis, there is a problem in processing speed and it is impossible to diagnose a large amount of sensor data. Recent studies use neural network models to diagnose a vehicle, but they diagnose only specific parts, not the overall condition of the vehicle. In addition, special hardware equipment is required to run neural network models in the vehicle in real time.

This paper presents the following main functions to solve these problems. First, the multi-protocol integrated gateway module (MIGM) in the lightweight autonomous vehicle self-diagnosis (LAVS) acts as an internal gateway to existing vehicles, ensures the traffic of vehicle communication in real time, and manages sensor messages for vehicle diagnosis. Second, to improve autonomous vehicle processing speed and accuracy, the in-vehicle diagnosis module (In-VDM) in the LAVS combines a random forest with a neural network model. It not only diagnoses the parts of a vehicle by learning a random forest model, but also the total condition of a vehicle by learning a neural network model. Therefore, the LAVS can reduce the overload of vehicle-to-cloud(V2C) communication because it delivers only diagnostic results to the Cloud and improve the safety of an autonomous vehicle. 

The composition of this paper is as follows. Section 2 describes the existing studies related to the LAVS in this paper. Section 3 details the structure and operation of the LAVS. Section 4 compares it with the existing methods to analyze the performance. Section 5 discusses the conclusion of the proposed LAVS and future research directions.

## 2. Related Works

### 2.1. Gateway

Vehicle gateways are the most important technology in autonomous vehicles. Basically, this not only enables communication between the server and the sensor but also leads to a serious accident if the communication environment of an autonomous vehicle becomes unstable. Therefore, researches on automotive gateways in various fields are actively underway.

Radier et al. proposed a vehicle gateway (VGW) that is auto-configured by the network and that eases mobility management. Indeed, the high mobility of a vehicle may imply many changes of access points; nevertheless, the connection must not be broken even during these access point changes. The connection to the new access point must be authorized and the VGW authenticated before leaving the communication range of the last access point. It also introduces a knowledge plane. The role of the knowledge plane is to know the environment of the user, to define the better link to use and control the mobility of the VGW introducing also new architecture to securely bind the user to the IP multimedia subsystem (IMS) [2].

Xie et al. examines in detail the various actual arriving orders of gateway messages and analyzes the real-time property of such in-vehicle networks. Worst case response time (WCRT) analysis for gateway-integrated controller area network (CAN) messages has been used. In this study, experimental results for a real message set demonstrates as much as 24% reduction of WCRT, compared with those obtained using the state-of-the-art methods [3].

Kim et al. presents a FlexRay-CAN gateway using a node-mapping method. The provided gateway describes the operating algorithm. In addition, it also solves the problem of having to reload the modified message mapping table on the gateway if the network message ID change and software complexity changes. This gateway solves three problems. First, when the message ID for the network is changed, the gateway must be reloaded with the revised message-mapping table. Second, if the number of messages exchanged is increased in the network, the complexity of the gateway software rapidly increases. In order to overcome these obstacles, this paper presents a FlexRay-CAN gateway using a node-mapping method. A gateway operation algorithm is described, and an experimental evaluation for ID change and software complexity is presented [4].

Kim et al. proposes a gateway framework for in-vehicle networks (IVNs) based on the controller area network (CAN), FlexRay, and Ethernet. The proposed gateway framework is designed to be easy to reuse and verify to reduce development costs and time. The gateway framework can be configured, and its verification environment is automatically generated by a program with a dedicated graphical user interface (GUI). The gateway framework provides state-of-the-art functionalities that include parallel reprogramming, diagnostic routing, network management (NM), dynamic routing update, multiple routing configuration, and security [5].

Benslimane envisions a vehicular ad hoc network (VANET)-universal mobile telecommunication system (UMTS) integrated network architecture. In this architecture, vehicles are dynamically clustered according to different related metrics. From these clusters, a minimum number of vehicles, equipped with IEEE 802.11p and UTRAN interfaces, are selected as vehicular gateways to link VANET to UMTS. Issues pertaining to gateway selection, gateway advertisement and discovery, and service migration between gateways (i.e., when serving gateways lose their optimality) are all addressed and an adaptive mobile gateway management mechanism is proposed [6].

Omar et al. introduces a new strategy for deploying internet gateways on the roads, together with a novel scheme for data packet routing, in order to allow a vehicle to access the internet via multi-hop communications in a VANET. The gateway placement strategy is to minimize the total cost of gateway deployment, while ensuring that a vehicle can connect to an internet gateway (using multi-hop communications) with a probability greater than a specified threshold. This cost-minimization problem is formulated using binary integer programming, and applied to a realistic city scenario, consisting of the roads around the University of Waterloo, Waterloo, ON, Canada. On the other hand, the developed packet routing scheme is based on a multichannel medium access control protocol, known as VeMAC, using time division multiple access. The performance of this cross-layer design is evaluated for a multichannel VANET in a highway scenario, mainly in terms of the end-to-end packet delivery delay. The end-to-end delay is calculated by modeling each relay vehicle as a queuing system, in which the packets are served in batches of no more than a specified maximum batch size [7].

Kaveh et al. proposed the connectivity-aware minimum-delay geographic routing (CMGR) protocol for vehicular ad hoc networks (VANETs), which adapts well to continuously changing network status in such networks. When the network is sparse, CMGR takes the connectivity of routes into consideration in its route selection logic to maximize the chance of packet reception. On the other hand, in situations with dense network nodes, CMGR determines the routes with adequate connectivity and selects among them the route with the minimum delay. The performance limitations of CMGR in special vehicular networking situations are studied and addressed. These situations, which include the case where the target vehicle has moved away from its expected location and the case where traffic in a road junction is so sparse that no next-hop vehicle can be found on the intended out-going road, are also problematic in most routing protocols for VANETs. Finally, the proposed protocol is compared with two plausible geographic connectivity-aware routing protocols for VANETs, A-STAR, and vehicle-assisted data delivery (VADD) [8].

Maurizio et al. proposed a new combinatorial optimization problem that arises from the framework of rule-based risk mitigation policies for the routing of gateway location problem (GLP) hazardous materials vehicles. GLP consists of locating a fixed number of check points (so called gateways) selected out of a set of candidate sites and routing each vehicle through one assigned gateway in such a way that the sum of the risks of vehicle itineraries is minimized. This paper addresses a GLP preparatory step, that is, how to select candidate sites, and it investigates the impact of different information guided policies for determining such a set. All policies consist of selecting a ground set and sampling it according to a probability distribution law. A few criteria are proposed for generating ground sets as well as a few probability distribution laws. A deterministic variant based on a cardinality constrained covering model is also proposed for generating candidate site sets [9].

**Figure 1:**
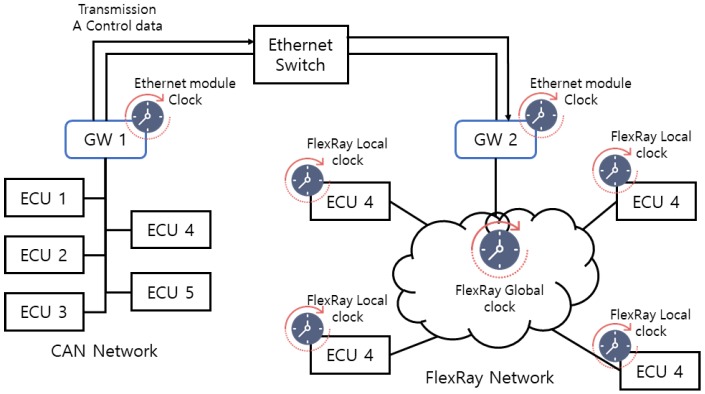
The communication between the controller area network (CAN) and FlexRay.

Lee et al. proposes a synchronization mechanism for FlexRay and Ethernet audio video bridging (AVB) network that guarantees a high quality-of-service. Moreover, this study uses an in-vehicle network environment that consists of FlexRay and Ethernet AVB networks using an embedded system, which is integrated and synchronized by the gateway. The synchronization mechanism provides the timing guarantees for the FlexRay network that are similar to those of the Ethernet AVB network. Figure 1 shows the use of the Ethernet AVB switch to communicate between the event-based CAN protocol and the timing-based FlexRay protocol [10].

Aljeri et al. proposed a reliable quality of service (QoS) aware and location aided gateway discovery protocol for vehicular networks by the name of fault tolerant location-based gateway advertisement and discovery. One of the features of this protocol is its ability to tolerate gateway routers and/or road vehicle failure. Moreover, this protocol takes into consideration the aspects of the QoS requirements specified by the gateway requesters; furthermore, the protocol insures load balancing on the gateways as well as on the routes between gateways and gateway clients [11].

Duan et al. proposed software defined networking (SDN) enabled 5G VANET. With proposed dual cluster head design and dynamic beamforming coverage, both trunk link communication quality and network robustness of vehicle clusters are significantly enhanced. Furthermore, an adaptive transmission scheme with selective modulation and power control is proposed to improve the capacity of the trunk link between the cluster head and base station. With cooperative communication between the mobile gateway candidates, the latency of traffic aggregation and distribution is also reduced [12]. 

Ju et al. proposed a novel gateway discovery algorithm for VANETs, providing an efficient and adaptive location-aided and prompt gateway discovery mechanism (LAPGD). Here, all vehicles go across selected mobile gateways to access 3G networks instead of a direct connection. The algorithm aims to ensure every vehicle is capable of finding its optimal gateway, to minimize the total number of gateways selected in VANETs, and to guarantee the average delay of packets within an allowable range [13].

Jeong et al. proposed “An Integrated Self-diagnosis System (ISS) for an Autonomous Vehicle based on an Internet of Things (IoT) Gateway and Deep Learning”. The ISS collects data from the sensors of a vehicle, diagnoses the collected data, and informs the driver of the result. The ISS considers the influence between its parts by using deep learning when diagnosing the vehicle. By transferring the self-diagnosis information and by managing the time to replace the car parts of an autonomous driving vehicle safely, ISS reduces loss of life and overall cost [14]. They proposed “A Lightweight In-Vehicle Edge Gateway (LI-VEG)” for the self-diagnosis of an autonomous vehicle. LI-VEG supports a rapid and accurate communication between in-vehicle sensors and a self-diagnosis module and between in-vehicle protocols. The LI-VEG has higher compatibility and is more cost effective because it applies a software gateway to the OBD, compared to a hardware gateway. In addition, it can reduce the transmission error and overhead caused by message decomposition because of a lightweight message header [15].

### 2.2. Random-Forest

**Figure 2:**
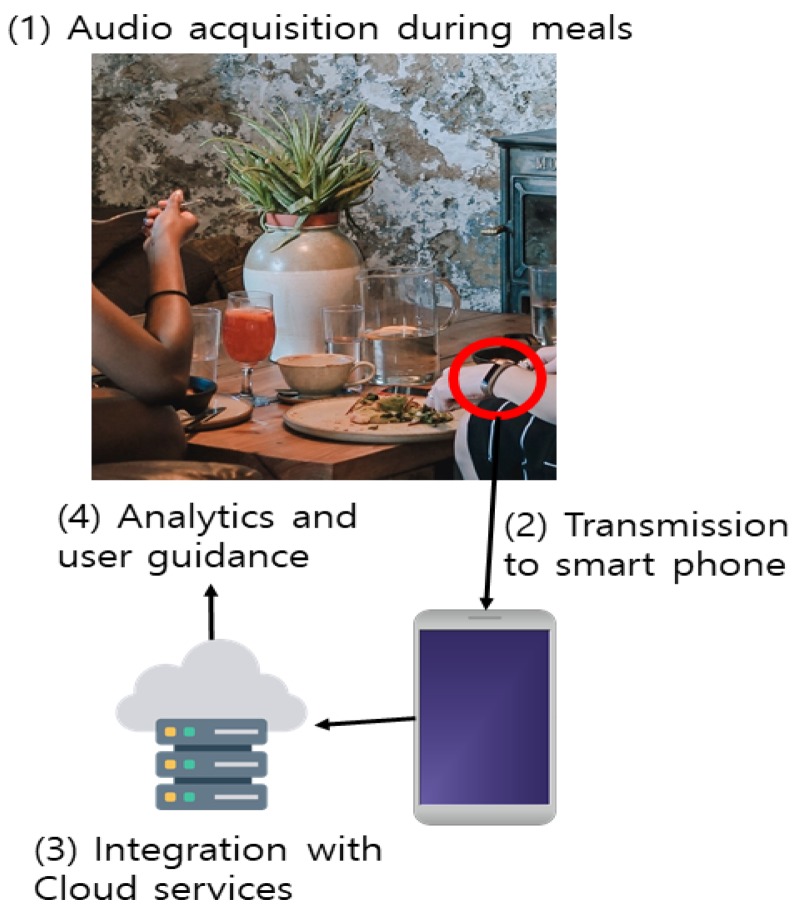
A high level architecture of the proposed system.

Mu used a random forest algorithm to increase customer loyalty through investigations of customer statistics, and dynamic and enterprise service attributes. As a result, we have taken appropriate steps to improve the accuracy of our forecasts of customer loyalty and to prevent customer losses [16]. Figure 2 shows high level architecture of the proposed system. Though identifying eating-related gestures using wrist-worn devices is a viable application of the watch, the focus of our work is to explore the idea of using audio to detect eating behavior based on bites, rather than swallows as other works have done.

Kalantarian et al. described signal-processing techniques for identification of chews and swallows using smart watch devices built-in microphone. In addition, the goal is to evaluate the potential of smartwatches as a platform for nutrition monitoring. Thus, signal processing technology uses random forest classifiers to classify sounds in a given environment. Random forests classify sounds based on a certain number of samples [17].

Huang et al. proposed a classification algorithm based on local cluster centers (CLCC) for data sets with a few labeled training data. The experimental results on uci data sets show that CLCC achieves competitive classification accuracy as compared to other traditional and state-of-the-art algorithms, such as sequential minimal optimization (SMO), adaptive boosting (AdaBoost), random tree, random forest, and co-forest [18].

Kalantarian et al. proposed a probabilistic algorithm for segmenting time-series signals, in which window boundaries are dynamically adjusted when the probability of the correct classification is low. Time-series segmentation refers to the challenge of subdividing a continuous stream of data into discrete windows, which are individually processed using statistical classifiers to recognize various activities or events. The algorithm improves the number of correctly classified instances from a baseline of 75%–94% using the random forest classifier [19].

Tahani et al. proposed the three data mining algorithms, namely the self-organizing map (SOM), C4.5, and random forest. They are applied on adult population data from the Ministry of National Guard Health Affairs (MNGHA), Saudi Arabia to predict diabetic patients using 18 risk factors. Health care data is often huge, complex, and heterogeneous because it contains different variable types and missing values as well. Therefore, data extraction using data mining was applied. Random forest achieved the best performance compared to other data mining classifiers [20].

AI-Jarrah et al. proposes a semi-supervised multi-layered clustering model (SMLC) for network intrusion detection and prevention tasks. SMLC has the capability to learn from partially labeled data while achieving a detection performance comparable to that of the supervised Machine Learning (ML)-based intrusion detection and prevention system (IDPS). The performance of the SMLC is compared with a well-known semi-supervised model (i.e., tri-training) and supervised ensemble ML models, namely, random forest, bagging, and AdaboostM1 on two benchmark network intrusion datasets, the NSL and Kyoto 2006+. In addition, SMLC demonstrates detection accuracy comparable to that of the supervised ensemble models [21].

Meeragandhi et al. evaluates the performance of a set of classifier algorithms of rules (JRIP, decision table, PART, and OneR) and trees (J48, random forest, REP Tree, and NB Tree). Based on the evaluation results, the best algorithms for each attack category are chosen and two classifier algorithm selection models are proposed. The classification models used the data collected from knowledge discovery databases (KDD) for intrusion detection. The trained models were then used for predicting the risk of the attacks in a web server environment or by any network administrator or any security experts [22].

Huang et al. proposes an approach to diagnose broken rotor bar failure in a line start-permanent magnet synchronous motor (LS-PMSM) using random forests. The transient current signal during the motor startup was acquired from a healthy motor and a faulty motor with a broken rotor bar fault. He extracted 13 statistical time domain features from the startup transient current signal, and used these features to train and test a random forest to determine whether the motor was operating under normal or faulty conditions. For feature selection, the feature importances from the random forest were used to reduce the number of features to two features. The results showed that the random forest classifies the motor condition as healthy or faulty with an accuracy of 98.8% using all features and with an accuracy of 98.4% using only the mean-index and impulsion features. This approach can be used in the industry for online monitoring and fault diagnostic of LS-PMSM motors and the results can be helpful for the establishment of preventive maintenance plans in factories [23].

## 3. A Design of the Lightweight Autonomous Vehicle Self-Diagnosis (LAVS)

### 3.1. Overview

**Figure 3:**
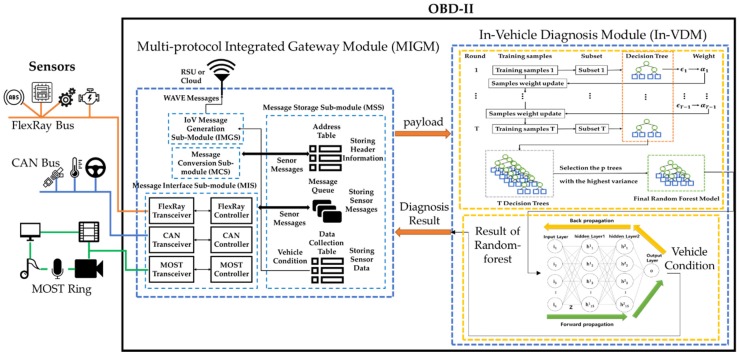
The structure of the lightweight autonomous vehicle self-diagnosis (LAVS).

In this section, Figure 3 shows the structure of the LAVS, which has two key modules in this paper: Multi-protocol integrated gateway module (MIGM) and in-vehicle diagnosis module (In-VDM). First, the MIGM supports communication between the internal protocols of a vehicle and transmits the payloads of sensor messages collected from the vehicle to the In-VDM. To improve the accuracy and processing speed of vehicle diagnosis, the In-VDM applies the random forest to the part self-diagnosis and the neutral network to the total self-diagnosis. It performs the part diagnosis of the vehicle itself independently of the Cloud and uses the results of this part diagnosis as an input for the total diagnosis of the vehicle.

### 3.2. The Multi-Protocol Integrated Gateway Module (MIGM)

**Figure 4:**
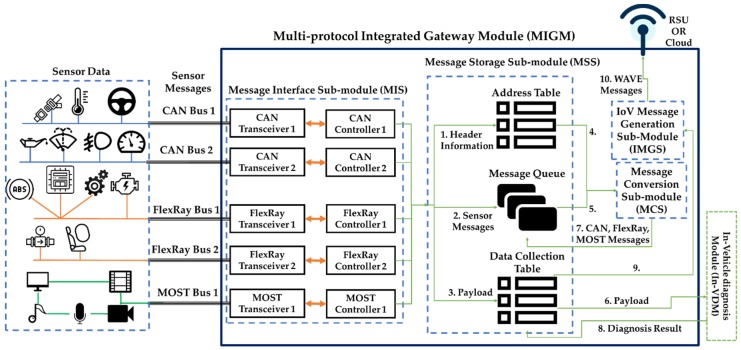
The structure of the multi-protocol integrated gateway module (MIGM).

Since the MIGM supports the in-vehicle communication between two protocols, transfers the payloads to the In-VDM rapidly, and works in the OBD-II in software, not in hardware, it improves the speed of the self-diagnosis. Figure 4 shows the structure and functions of the MIGM, which consists of four sub-modules. The first message interface sub-module (MIS) acts as an interface between the sensors and the MSS. The second message storage sub-module (MSS) manages the message transferred from the MIS and the message converted in the MCS. The third message conversion sub-module (MCS) converts the message transferred from the MSS to a destination protocol message. The fourth WAVE message generation sub-module (WMGS) makes the vehicle condition diagnosed in the In-VDM and the payloads used for the diagnosis a WAVE message and transfers the WAVE message to RSU or the Cloud through the internet of vehicles (IoV). If the MIGM receives a sensor message from electronic control unit (ECU), the message works as follows.

First, the ECU transmits the sensor messages to hardware devices (transceiver and controller) through FlexRay, CAN, and media oriented systems transport (MOST) Bus. Second, the MIS transfers the sensor messages to the MSS. Third, the MSS divides the received sensor messages into header information, sensor messages, and payloads. The received header information is stored in an address table (1), the sensor messages are stored in a message queue (2), and the payloads are stored in a data collection table (3). The MSS transfers the header information of the address table and the sensor messages to the MCS (4, 5), and the payloads measured in the data collection table in the same time to the In-VDM(6). Fourth, the MCS converts the sensor message transferred from the MSS to a destination protocol message and transmits the transformed message to the MSS (7) and, the process of the message reception is vice versa. Fifth, if the MSS of the MIGM receives the diagnosis result from the In-VDM, the received result is stored in the data collection table (8). The self-diagnosis data stored in it is transferred to the WMGS with the payloads used for self-diagnosis (9). The WMGS converts the received data collection table information to WAVE messages and performs IoV to transfer the WAVE messages to the neighboring RSU and Cloud (10).

#### 3.2.1. A Design of a Message Interface Sub-Module (MIS)

**Figure 5:**
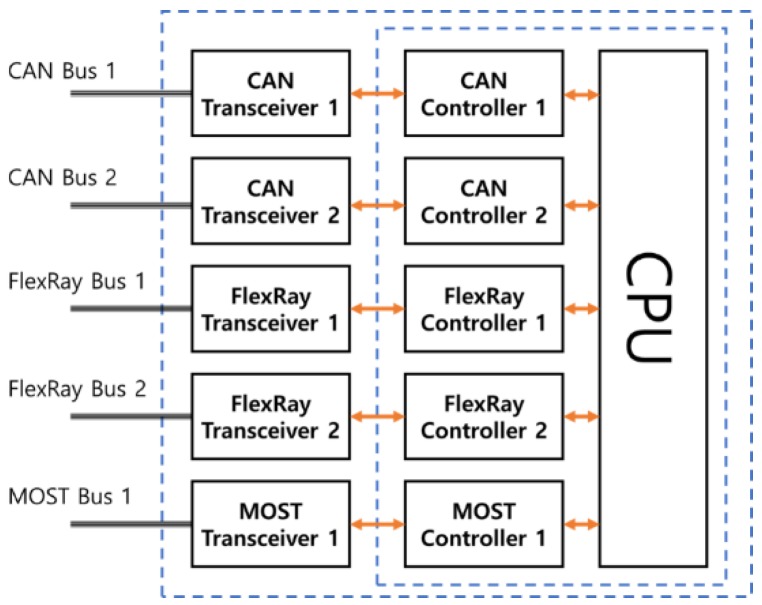
The hardware structure of the MIGM.

The MIS acts as an interface between hardware and the MSS. The hardware means the transceiver and controller sending messages to and receiving them from each protocol bus. If the transceiver receives messages from each protocol bus or an actuator and transfers the received messages to a controller, the controller stores the serial bits of messages in the MCU. The MIS transfers the messages of a controller to the MSS. Figure 5 shows the hardware structure of a transceiver and controller.

If the message translation is completed, the message has to be transferred to a destination bus. At this time, the MIS receives the messages translated in the MSS and transfers them to the controller. The controller delivers the messages to a protocol bus or an actuator through the transceiver.

#### 3.2.2. A Design of a Message Storage Sub-Module (MSS)

The MSS manages the messages received from the MIS. Figure 4 shows three functions (address table, message queue, and data collection table) in which the MSS manages messages as follows. 

**Figure 6:**
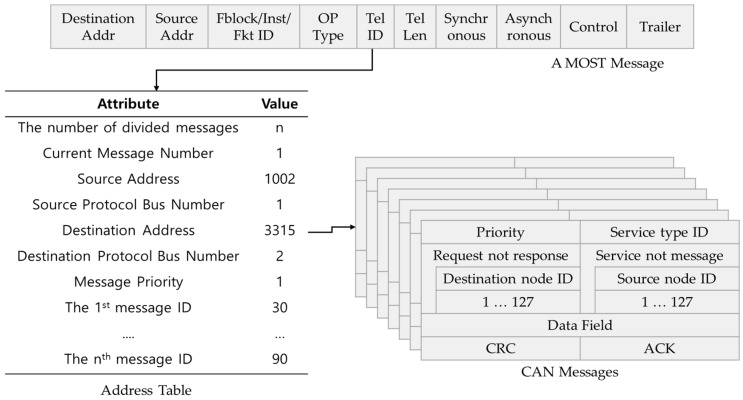
The conversion of a MOST message to CAN messages.

First, the header information (destination address, source address, etc.) is stored in the address table. When sensor messages are converted in the MCS, the address table helps them be converted rapidly and is used to detect errors. Figure 6 shows the address table in detail. 

Second, the message queue consists of an input message queue and an output message queue. The input message queue is one that stores the messages transferred from the MIS. The MSS transfers the messages to the MCS according to message order within the queue. The order of messages within the input message queue is decided by the priority of messages. The output message queue is one storing the messages transferred from the MCS and the MSS transfers the messages of the output message queue to the MIS according to priority. 

Third, the payloads of the received sensor messages are stored in the data collection table and then they are transferred to the IN-VDM. If the IN-VDM completes the self-diagnosis of an autonomous vehicle, the MSS stores the diagnosed result in the data collection table. The payloads and vehicle diagnosis result collected in the same hour are transferred to the MCS and the MCS converts the collected payloads and vehicle diagnosis result to a WAVE message. The WAVE messages are transferred to the neighboring RSU and the Cloud through IoV. Table 1 shows the example of the data collection table. 

The payloads (or data) of Table 1 are collected within 0.5 sec and the diagnosed result by the In-VDM is made based on the payloads. The payloads and the diagnosed result are transferred to the MCS and are converted to WAVE messages. The generation process of a WAVE message is shown in Section 3.2.3 in detail.

Totally, the MSS manages the received messages, transfers the header information of the address table and the messages of the message queue to the MCS and transfers to the MIS the messages converted in the MCS.

#### 3.2.3. A Design of the Message Conversion Sub-Module (MCS)

The MCS converts sensor messages to the message type of the other protocol by receiving the messages of the input message queue and the address information of the address table in the MSS. When the messages are converted, the MCS uses the address information of the address table. The MIGM maps rapidly to the message fields of the other protocol the values of the address table, which is generated by each protocol. For example, the address tables used between protocols such as FlexRay to CAN, MOST to FlexRay, etc. are generated separately. The field values of the address table are generated newly whenever messages are converted. Table 2 shows the example of the address table used when MOST to CAN conversion is done. 

When MOST messages are converted to CAN messages, the attributes of the address table are not changed, but their values are changed according to the message state. Figure 6 shows the conversion of a MOST message to CAN messages by using the address table of Table 2. 

In Figure 6, the field of Destination Addr(address) in a MOST message is mapped to the destination node IDs of CAN messages through the field of destination address in the Address Table and the field of Source Addr(address) in a MOST message is mapped to the source node IDs of CAN messages through the field of source address in the Address Table. Since the Fblock/Inst/Fkt ID and OP Type field of a MOST message are not used in CAN messages, they are not stored in the address table. The Tel(telephone) ID field of a MOST message is mapped to the service type IDs of CAN messages through the current message number and message ID field of the address table. The Synchronous and Asynchronous field of a MOST message is used for MOST and they are converted to the data field of CAN messages without using the address table. Since the Control and Trailer field of a MOST message is not used for CAN messages, they are not converted. The CAN messages generate the cyclic redundancy check(CRC) and acknowledgement(ACK) field of CAN messages for themselves.

#### 3.2.4. A Design of a WAVE Message Generation Sub-Module (WMGS)

The WMGS generates a WAVE message after receiving information from the data collection table in the MSS.

**Figure 7:**
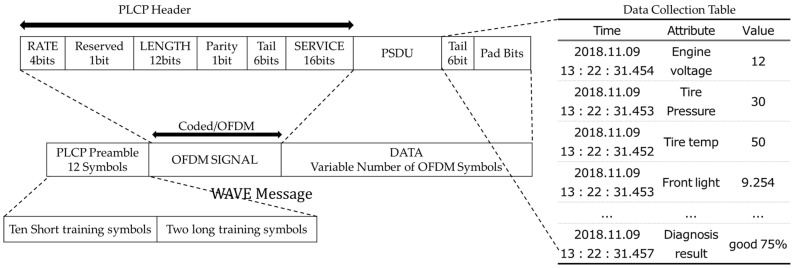
The generation of a WAVE message using the data collection table.

Figure 7 shows the structure of a generated WAVE message. Since the WAVE message is not converted to a message type of other protocols, it has to set message values newly. The physical layer convergence protocol (PLCP) preamble of the WAVE message consists of the same 10 short training symbols and two long training symbols. The PLCP preamble uses the same bits as that of the Ethernet. Since the WAVE message uses an orthogonal frequency division multiplexing (OFDM), it needs a OFDM signal field whose RATE means a frequency division rate. The frequency division rate is decided according to the size of a message. The reserved of the OFDM signal field represents an address that receives messages. The LENGTH of the OFDM signal field represents the length of a message. The Parity of the OFDM signal field is used to examine errors and the Tail of the OFDM Signal field means the end of the OFDM signal field. The DATA field consists of a service field, a PLCP service data unit(PSDU) field meaning data, a tail field meaning message end, and pad bits examining the error of the DATA field [24]. After the payloads and diagnosed result measured in the same time are converted to the PSDU of the WAVE message, the WMGS transfers the converted message to the neighboring RSU or the Cloud.

### 3.3. A Design of an In-Vehicle Diagnosis Module (In-VDM)

**Figure 8:**
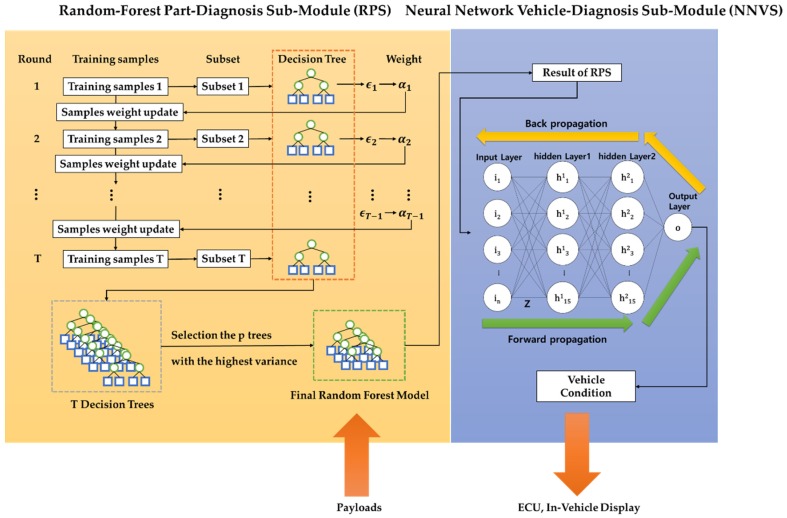
The structure of the in-vehicle diagnosis module (In-VDM).

Figure 8 shows the structure of the In-VDM, which consists of two sub-modules. The first random-forest part-diagnosis sub-module (RPS) generates a random-forest model for each part of a vehicle and diagnoses the parts of a vehicle by using the generated random-forest model. The second neural network vehicle-diagnosis sub-module (NNVS) generates a neural network model and diagnoses the total condition of the vehicle by using the results of the RPS as input.

#### 3.3.1. A Design of the Random-Forest Part-Diagnosis Sub-Module (RPS)

The RPS learns a random-forest model using training data and outputs conditions by part. Algorithm 1 shows the process by which the RPS generates a random-forest model.

**Algorithm 1.** The process generating a random-forest model.Input: Training data X, Y, W X = set of payloads Y = set of results of training data W = set of weights   initialize weight W : w_i_^(1)^ = 1/N for(int j = 1; j <= T; j++) make subset S_t_ from Training data. ΔGmax = -∞ sample feature f from sensors randomly for(int k = 1; k <= K; k++) S_n_ = a current node split S_n_ into S_l_ or S_r_ by f_k_ compute information gain ΔG: $\Delta G=E\left(S_{n}\right)-\frac{\left|S_{l}\right|}{\left|S_{n}\right|}E\left(S_{l}\right)-\frac{\left|S_{r}\right|}{\left|S_{n}\right|}E\left(S_{r}\right)$ if(ΔG>ΔGmax) ΔGmax = ΔG end if end for if(ΔGmax = 0 or maximum depth) store the probability distribution P(c|l) in a leaf node. else generate a split node recursively. end if if(finish training of decision tree) estimate class label : $\hat{y_{i}}$ $\hat{y_{i}}$ = arg max Pt(c|l). compute an error rate of a decision tree : $\epsilon_{t}=\sum_{i:y_{i}\neq \hat{y_{i}}}^{N}w_{i}^{\left(t\right)}/\sum_{i=1}^{N}w_{i}^{\left(t\right)}$
 compute a weight of a decision tree : $\alpha_{t}=\frac{1}{2}log\frac{1-\epsilon_{t}}{\epsilon_{t}}$
 if($\alpha_{t}$ > 0 then) update a weight of training data  $w_{i}^{\left(t+1\right)}=\{\begin{array}{c} if\left(y_{i}\neq \hat{y_{i}}\right)\;w_{i}^{\left(t\right)}\exp\left(\alpha_{t}\right)\\ else\;w_{i}^{\left(t\right)}\exp\left(-\alpha_{t}\right) \end{array}$ else reject a decision tree end if end if end for

The training data composed of X, Y, and W. X is the set of payloads and is represented in the following formula (1).

(1) $$X=\left\{x_{1},x_{2},\;\ldots ,x_{N}\right\}.$$

In Formula 1, *X* means sensor data configured for each part. For example, $X_{\mathrm{Engine}}$ is a set of payloads that can represent the engine condition. *Y* means the result value judging whether a part condition is normal or not. *Y* consists of parts, as in *X*. It has one between a normal value and an abnormal value in the following formula (2).

(2) $$Y=\left\{y_{1},y_{2},y_{3},y_{4},\;\ldots ,\;y_{N}\right\}.$$

*W* means the weight of each training data and is represented in the following formula (3).

(3) $$W=\left\{w_{1},w_{2},w_{3},w_{4},\;\ldots ,\;w_{N}\right\}.$$

All *w_i_* is initialized as 1/N at the beginning. Here, *N* represents the number of training data. The RPS generates a decision-making tree by extracting the variables according to weight and composes a random-forest model by modifying each variable and the tree weight. The RPS generates decision-making trees composing a random-forest model. The decision-making tree is made by using the information gain function, ΔG computed by using Genie function. The RPS repeats itself until the decision-making tree reaches a fixed depth or until the ΔG becomes 0. The decision-making trees are generated as follows. 

First, ΔG_max_ is set as −∞ for the decision-making tree generation and the decision-making tree generation sub-module generates subset, S_t_ from the training data. 

Second, it selects one of all sensors randomly. 

Third, it classifies the training data S_n_ of a current node into *S_l_* and *S_r_* and computes the ΔG in formula (4).

(4) $$\Delta G=G\left(S_{n}\right)-\frac{\left|S_{l}\right|}{\left|S_{n}\right|}G\left(S_{l}\right)-\frac{\left|S_{r}\right|}{\left|S_{n}\right|}G\left(S_{r}\right),$$

where, *S_l_* is the value of a left child node in the S_n_ and *S_r_* is the value of a right child node in the S_n_. G(s) as the Genie index is computed in formula (5).

(5) $$G\left(s\right)=1-\sum_{j=1}^{2}P{\left(c_{j}\right)}^{2}.$$

In formula (5), the probability, $P\left(c_{j}\right)$ of the node *c_j_* is computed in formula (6).

(6) $$P\left(c_{j}\right)=\sum_{i\in S\wedge y_{i}=c_{j}}w_{i}÷\sum_{i\in S}w_{i},$$

In formula (6), *w* means a weight of each variable.

**Figure 9:**
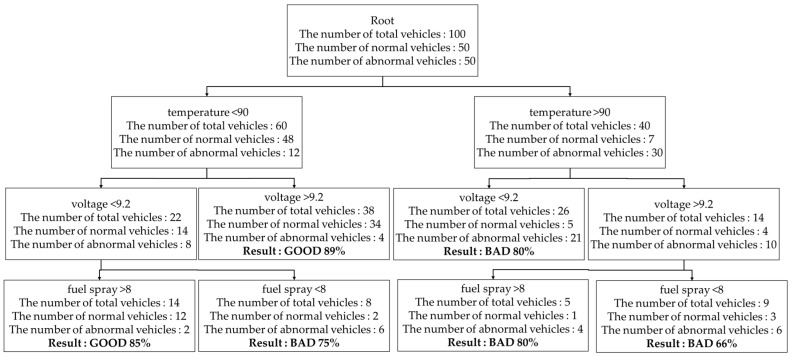
The decision-making tree generated based on temperature, voltage, and fuel spray.

Figure 9 shows that a decision-making tree was generated by using three variables as follows.

First, 50 payloads of a normal vehicle and 50 payloads of an abnormal vehicle as training data was used for the RPS and temperature, fuel spray, and voltage were selected as a variable. 

Second, the RPS computes the Genie index with total training data, each variable, and information gain ΔG. The computation is done as follows.

At the beginning, the Genie index of training data without classification criteria has to be measured only once. The actual value of the Genie index is obtained as follows.

$$G\left(\mathrm{root}\right)=1-{\left(\frac{50}{100}\right)}^{2}-{\left(\frac{50}{100}\right)}^{2}=0.5$$

If the Genie index of the training data is obtained without the classification criteria, the Genie index of each variable is obtained with the classification criteria. The following formulas are used to measure the Genie index when the condition of a vehicle is classified according to temperature classification criteria.

$$G\left(S_{\mathrm{temp}\;l}\right)=1-{\left(\frac{48}{60}\right)}^{2}-{\left(\frac{12}{60}\right)}^{2}=0.32$$

$$G\left(S_{\mathrm{temp}\;r}\right)=1-{\left(\frac{7}{40}\right)}^{2}-{\left(\frac{33}{40}\right)}^{2}=0.28$$

Here, the number 60 used as the denominator in the $G\left(S_{\mathrm{temp}\;l}\right)$ formula means the 60 of 100 vehicles whose temperature numeric is normal. The 48 used as the numerator in the $G\left(S_{\mathrm{temp}\;l}\right)$ formula means the 48 of 60 vehicles whose total condition is normal and the 12 means the 12 of 60 vehicles whose total condition is abnormal. The 40 used as denominator in the $G\left(S_{\mathrm{temp}\;r}\right)$ formula means the 40 of 100 vehicles whose temperature numeric is abnormal. The seven used as the numerator in the $G\left(S_{\mathrm{temp}\;r}\right)$ formula means the seven of 40 vehicles whose total condition is normal and the 33 means the 33 of 40 vehicles whose total condition is abnormal. In the same way, the RPS computes the Genie index of the fuel spray and voltage as follows.

$$G\left(S_{\mathrm{fuel}\;l}\right)=1-{\left(\frac{25}{55}\right)}^{2}-{\left(\frac{30}{55}\right)}^{2}=0.49$$

$$G\left(S_{\mathrm{fuel}\;r}\right)=1-{\left(\frac{26}{45}\right)}^{2}-{\left(\frac{19}{45}\right)}^{2}=0.49$$

$$G\left(S_{V\;l}\right)=1-{\left(\frac{48}{70}\right)}^{2}-{\left(\frac{22}{70}\right)}^{2}=0.43$$

$$G\left(S_{V\;r}\right)=1-{\left(\frac{10}{30}\right)}^{2}-{\left(\frac{20}{30}\right)}^{2}=0.44$$

If the computation of the Genie index of each variable is finished, the RPS computes the information gain ΔG of each variable as follows.

$$\Delta G_{\mathrm{temp}}=0.5-\frac{60}{100}*0.32-\frac{40}{100}*0.28=0.196$$

$$\Delta G_{\mathrm{fuel}}=0.5-\frac{55}{100}*0.49-\frac{45}{100}*0.49=0.01$$

$$\Delta G_{V}=0.5-\frac{70}{100}*0.43-\frac{30}{100}*0.44=0.067$$

Here, since the information gain about the temperature is the biggest value, the 1st classification node is decided as the temperature. Once the 1st classification node is decided, the RPS computes the Genie indices of the other variables again. The following formulas show that the Genie index of the fuel spray is computed after the vehicles were classified with the temperature.

$$G\left(S_{\mathrm{fuel}\;l}\right)=1-{\left(\frac{24}{29}\right)}^{2}-{\left(\frac{5}{29}\right)}^{2}=0.37$$

$$G\left(S_{\mathrm{fuel}\;r}\right)=1-{\left(\frac{26}{31}\right)}^{2}-{\left(\frac{7}{31}\right)}^{2}=0.24$$

The computed Genie index of the fuel spray has to be compared with the Genie index of the voltage under the same condition. The following formulas show that the Genie index of the voltage is computed after vehicles were classified with the temperature.

$$G\left(S_{V\;l}\right)=1-{\left(\frac{34}{38}\right)}^{2}-{\left(\frac{4}{38}\right)}^{2}=0.18$$

$$G\left(S_{V\;r}\right)=1-{\left(\frac{14}{22}\right)}^{2}-{\left(\frac{8}{22}\right)}^{2}=0.46$$

If the Genie index of the fuel spray and voltage is computed, the RPS computes the information gain on them. The following formulae are used to compute the information gain on them.

$$\Delta G_{\mathrm{fuel}}=0.32-\frac{29}{60}*0.37-\frac{31}{60}*0.24=0.017$$

$$\Delta G_{V}=0.32-\frac{38}{60}*0.18-\frac{22}{60}*0.46=0.037$$

Here, because the $\Delta G_{V}$ is the biggest value, the 2nd classification node is decided as the starting voltage. In this way, if the information gain is 0 or a decision-making tree reaches fixed depth, the decision-making tree is generated. 

**Figure 10:**
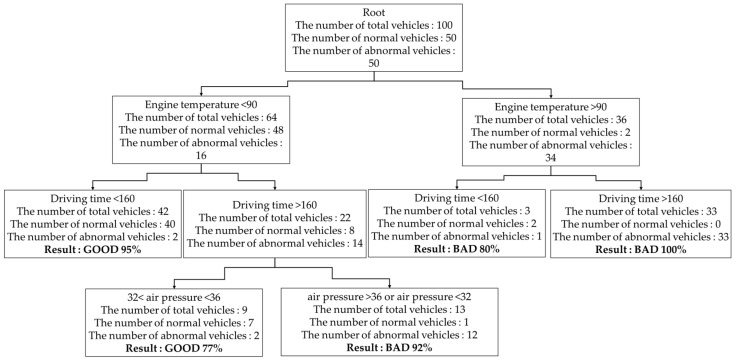
The decision-making tree generated based on the engine temperature, driving time, and air pressure.

Since the RPS extracts properties randomly whenever each decision-making tree is generated, all the different decision-making trees are generated. Figure 10 shows that the decision-making tree was generated with the variables different from Figure 9. The RPS computes a weight by using an error rate of a decision-making tree and generates a random-forest model based on boosting. First of all, in formula (7), the RPS computes the error rate ($\epsilon_{t}$) of the decision-making tree by comparing a diagnosed condition with the result of training data ($i:y_{i}\neq \hat{y_{i}}$).

(7) $$\epsilon_{t}=\sum_{i:y_{i}\neq \hat{y_{i}}}^{N}w_{i}^{\left(t\right)}/\sum_{i=1}^{N}w_{i}^{\left(t\right)},$$

where, $\hat{y_{i}}$ is the result of a random-forest model when training data is entered into the random-forest model. $y_{i}$ represents the vehicle condition stored in the training data. That is, $\epsilon_{t}$ is to divide the sum of weights in case of ($\hat{y_{i}}$ ≠ $y_{i}$) by total sum of weights. From now on, the RPS computes a weight change rate $\alpha_{t}$ by using formula (8).

(8) $$\alpha_{t}=\frac{1}{2}log\frac{1-\epsilon_{t}}{\epsilon_{t}}.$$

If the weight change rate was computed, the weight is modified in formula (9).

(9) $$w_{i}^{\left(t+1\right)}=\{\begin{array}{c} if\left(y_{i}\neq \hat{y_{i}}\right)\;w_{i}^{\left(t\right)}\exp\left(\alpha_{t}\right)\\ else\;w_{i}^{\left(t\right)}\exp\left(-\alpha_{t}\right) \end{array}.$$

**Figure 11:**
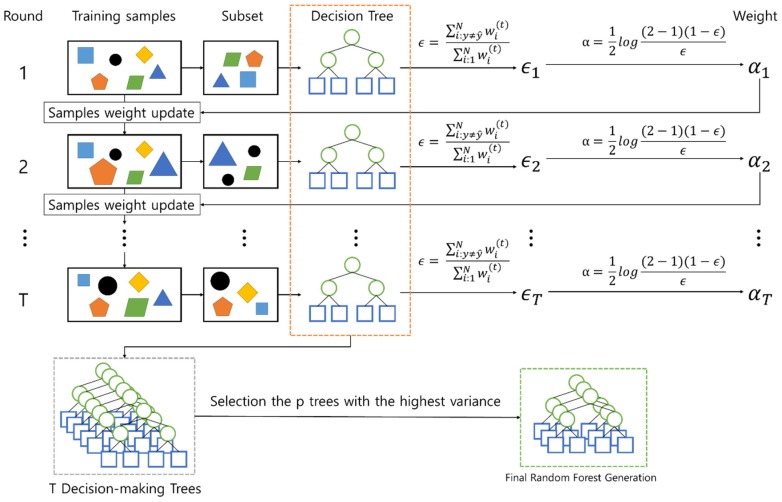
A random-forest model generated finally by boosting.

In Figure 11, if this process is repeated T times and T decision-making trees are generated, the RPS computes the variance of each tree and selects only p trees in ascending order of the variance value. The RPS generates the random-forest model with the selected p trees. Figure 10 shows how to compose T trees by using the weight of a decision-making tree. The former in Figure 10 generates a decision-making tree by sampling training data as a subset and the latter in Figure 10 modifies the weight by using an error rate ϵ and weight change rate α.

For example, to compute the weight the RPS computes an error rate of a tree through six terminal nodes in Figure 10.

$$\epsilon =\frac{2}{14}+\frac{5}{26}+\frac{3}{9}+\frac{1}{5}+\frac{2}{6}+\frac{4}{38}=0.11$$

Here, the denominator represents the number of total vehicles in a terminal node and the numerator is decided according to the result of a terminal node. If the result of the terminal node is ”GOOD”, the numerator represents the number of abnormal vehicles in the terminal node. If the result of the terminal node is “BAD”, the numerator represents the number of normal vehicles in the terminal node. For example, the $\frac{2}{14}$ represents an error rate of the leftmost terminal node in Figure 9. The denominator 14 represents the number of the total vehicles in the terminal node. Since the result of the terminal node is “GOOD”, the numerator 2 represents the number of abnormal vehicles and then the RPS computes a weight change rate α.

$$\alpha =\frac{1}{2}\log\frac{1-0.11}{0.11}=1.1091$$

The RPS modifies weight *w* with formula (9). Since the decision-making tree of Figure 9 was generated with 100 vehicles, the weight of 100 vehicles is set as 1/100 each. Therefore, the weight of 100 vehicles is modified according to the result of each terminal node and a weight change rate α.

$$w_{i}^{\left(2\right)}=\{\begin{array}{c} if\left(y_{i}\neq \hat{y_{i}}\right)\;0.01*e^{1.1091}=0.0303162\\ else\;0.01*e^{-1.1091}=0.0032985 \end{array}\left(i=1\sim 100\right).$$

If T trees are generated by repeating the processes, the RPS composes a random-forest model of only fixed trees by computing the variance value of trees.

The RPS generates the final random-forest model and computes the part condition by multiplying the probability, $P_{t}(c|x)$ of each decision-making tree by the decision-making tree weight, $\alpha_{t}$. Formula (10) is used to compute the vehicle condition, $\hat{y_{i}}$, in each decision-making tree.

(10) $$\hat{y_{i}}=\alpha_{t}*P_{t}(c|x).$$

If the probability of each decision-making tree is computed, the RPS selects the highest probability as the final probability, $\hat{y}$.

(11) $$\hat{y}=\mathrm{argmax}\left(\hat{y_{t}}\right).$$

After the RPS diagnosed this part, it informs drivers of a vehicle’s part condition and transfers the diagnosis result to the NNVS. 

#### 3.3.2. A Design of a Neural Network Vehicle-Diagnosis Sub-Module (NNVS)

**Figure 12:**
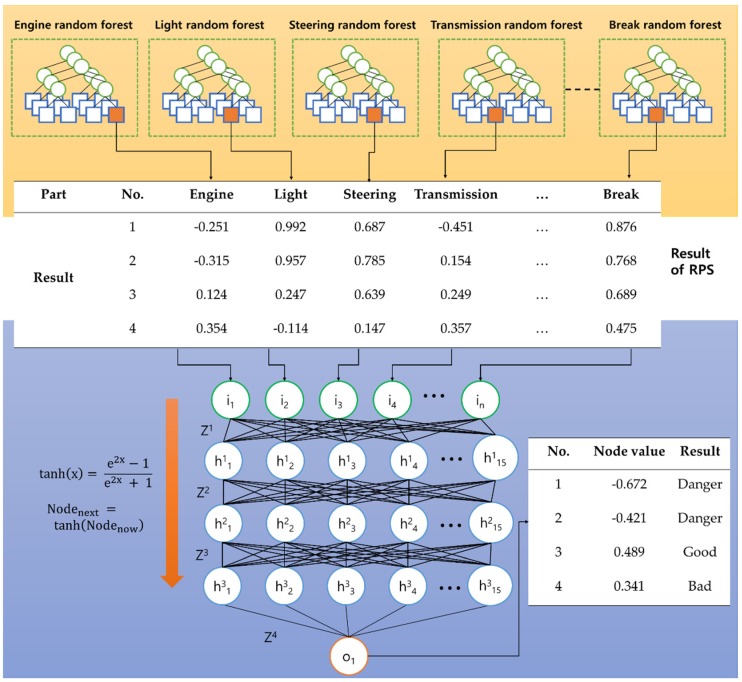
A process of the total diagnosis using the NNVS.

After the RPS diagnoses parts of a vehicle, the NNVS learns a neural network model using the result of the RPS and diagnoses the total condition of the vehicle by using the learned neural network model. Figure 12 shows an example of the input and output of the NNVS. 

The RPS result represents a probability value. However, it exists between −1 and 1 because the probability value is multiplied by −1 when the RPS determines that the part is abnormal. For example, in Table 3, the value of the engine condition for the RPS is 0.251, and because the engine has been diagnosed as faulty, −0.251 is delivered to the NNVP. Table 3 is used as an input to the NNVP in Figure 12. Algorithm 2 represents the process by which the NNVS learns neural networks. 

**Algorithm 2.** The learning process of the NNVSInput : Training data I, O I[] = result of RPS n = the number of input nodes y = training data initialize:   weight Z [3][][] :    for(int i = 0; i<n; i++){      for(int j = 0; j<15; j++){       Z [0][i][j] = sqrt(random(0,3)/n+15);    }   }   for(int i = 0; i<15; i++){      for(int j = 0; j<15; j++){       Z[1][i][j] = sqrt(random(0,3)/30);    }   }      for(int i = 0; i<15; i++){    Z[2][i][j] = sqrt(random(0,3)/16);   }   Emax = 0.03;   E = 900;   NET = 0;   H[2][15] = 0;   O = 0; while(E>Emax){ for(int i = 0; i<15; i++){  for(int j=0; j<n; j++){   NET = NET + I[j]*Z[0][j][i];  }  H[0][i] = tanh(NET);  NET = 0; } for(k = 1; k<3; k++){  for(int i = 0; i<15; i++){   for(int j = 0; j<15; j++){   NET = NET + H[k-1][j]*Z[k][j][i];   }   H[k][i] = tanh(NET);   NET = 0;  } }  for(int i = 0; i<15; i++){  NET = NET + H[2][i] * Z[3][i][0]; } O = tanh(NET);  E = pow((o-y),2); Update_wights(Z, E); }end

The NNVS uses the results of the RPS as an input, which indicates the condition of vehicle parts. That is, the number of the NNVS input nodes is equal to the number of the RPS outputs. The number of The NNVS output nodes is 1. Formula (12) represents the set of inputs in the NNVS, I.

(12) $$I=\left\{i_{1},\;i_{2},\ldots ,\;i_{n}\right\}.$$

Here, *n* is the number of parts diagnosed by the RPS. The NNVS generates a neural network model consisting of three hidden layers. Formula (13) represents the nodes of the hidden layers and Formula (14) represents a weight that connects the adjacent node.

(13) $$H=\left\{h_{1}^{1},{\;h}_{2}^{1},h_{3}^{1},h_{4}^{1},h_{5}^{1},\;\ldots ,{\;h}_{15}^{3}\right\},$$

(14) $$Z=\;\left\{z_{1,\;1}^{1},{\;z}_{1,\;2}^{1},z_{1,\;3}^{1},z_{1,\;4}^{1},z_{1,\;5}^{1},\;\ldots ,{\;z}_{15,\;15}^{3}\right\}.$$

The weights are initialized using the Xavier initialization [25]. Formula (15) represents the weights that are initialized using the Xavier initialization.

(15) $$z_{y,k}^{x}=\sqrt{\frac{random\left(0,3\right)}{p_{in}+p_{out}}},$$

Here, $p_{in}$ represents the number of nodes in the input layer connected to Z and $p_{out}$ the number of nodes in the output layer connected to Z. The NNVS uses tanh as an activation function for accurate and quick computation, and the mean squared error as a loss function. Formula (16) represents tanh and (17) mean squared error.

(16) $$tanh\left(x\right)=\frac{e^{2x}-1}{e^{2x}+1},$$

(17) $$E\left(y,\;d\right)={\left(y-d\right)}^{2}.$$

In Formula (17), d means the training data, and y means the result of a neural network model. The NNVS is learned based on back-propagation and uses gradient descent to change the weight.

When the NNVS completes its neural network model learning, it diagnoses the total condition of the vehicle by using the neural network model. The NNVS result of between 1 and 0.4 indicates that the vehicle is in good condition, that between 0.4 and −0.4 indicates that the vehicle is in a bad condition but capable of driving, and that between −0.4 and −1 indicates that the vehicle is in a dangerous condition. The NNVS delivers the results of the neural network model learning to the driver so that the driver can accurately understand the total condition of the vehicle.

## 4. The Performance Analysis

This section shows the performance analysis of the MIGM and the In-VDM. To analyze the performance of the MIGM, it was compared with an existing in-vehicle gateway to measure conversion time and an error rate when 4000 messages are converted to other messages and to analyze the performance of the IN-VDM, it was compared with a multi-layer perceptron (MLP) and a long short-term memory (LSTM) to measure the computation time and accuracy when the number of test data sets is changed.

### 4.1. The MIGM Performance Analysis

To compare the performance of the MIGM with an existing vehicle gateway, conversion time and an error rate are measured when 4000 messages are converted to other messages. The experiment was conducted when a CAN message was converted to a FlexRay or a MOST message and vice-versa. The existing in-vehicle gateway and the MIGM were implemented in the C language and the experiment was conducted in a Host PC.

**Figure 13:**
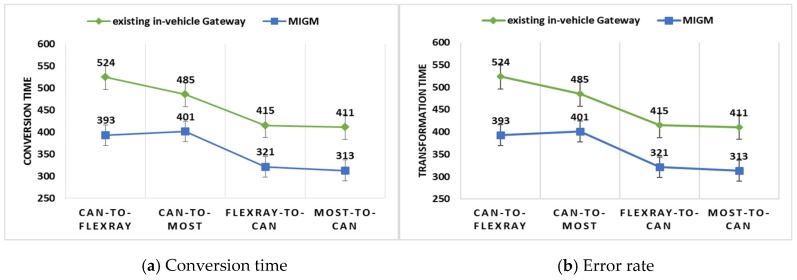
The (**a**) conversion time and (**b**) an error rate per protocol.

Figure 13a shows that in the CAN-To-FlexRay conversion, the MIGM was improved by 33.3% in conversion time more than the existing in-vehicle gateway, in the CAN-To-MOST conversion, the MIGM was improved by 20.9% more than the existing in-vehicle gateway, in FlexRay-To-CAN conversion, the MIGM was improved by 29.2% more than the existing in-vehicle gateway, and in the MOST-To-CAN conversion, the MIGM was improved by 31.3% more than the existing in-vehicle gateway. Therefore, the conversion time of the MIGM was improved more than that of the existing in-vehicle gateway by about average 28.67%.

Figure 13b shows that in the CAN-To-FlexRay conversion, the existing in-vehicle gateway causes an error rate, 1.55% and the MIGM an error rate, 1.55%, in the CAN-To-MOST conversion, the existing in-vehicle gateway causes an error rate, 1.66% and the MIGM an error rate, 0.45%, in FlexRay-To-CAN conversion, the existing in-vehicle gateway causes an error rate, 1.94% and the MIGM an error rate, 1.59%, and in the MOST-To-CAN conversion, the existing in-vehicle gateway causes an error rate, 2.59%, and the MIGM an error rate, 2.53%. Therefore, the error rate of the MIGM was lower than that of the existing in-vehicle gateway by about 0.5%. When a CAN message was converted to another protocol message, it had higher performance than other cases by 1%.

### 4.2. The In-VDM Performance Analysis

To analyze the performance of In-VDM, three experiments were conducted. In two experiments, NNVP was compared with multi-layer perceptron (MLP) and long short-term memory (LSTM) in computation time and accuracy, while in the other experiment, RPS was compared with a support vector machine (SVM) and fuzzy in test loss. According to the experimental environment, the number of test data sets was 100, 150, 200, 300, 400, and 500 sets, the computation speed was 3.20 GHz, and the RAM memory size was 16 GB.

**Figure 14:**
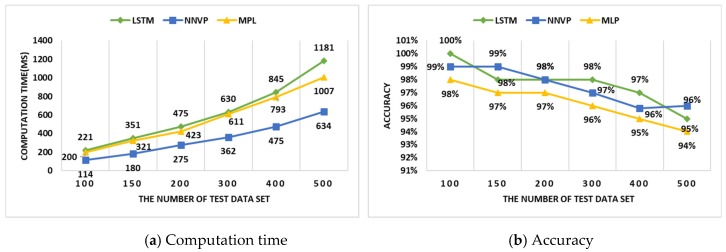
The (**a**) computation time and (**b**) accuracy according to test data.

Figure 14a shows that the computation time of the NNVP was improved by 44.894% and 62.719% more than that of the MPL and the LSTM separately as the number of test data sets increased from 100 to 500 sets. Figure 14b shows that the accuracy of the NNVP was higher by about 1% than that of the MLP but similar to that of the LSTM on average. Since there was little difference between them in accuracy but the NNVP was more efficient in computation time, NNVP was more suitable than the MPL and LSTM in vehicle self-diagnosis by using payloads.

**Figure 15:**
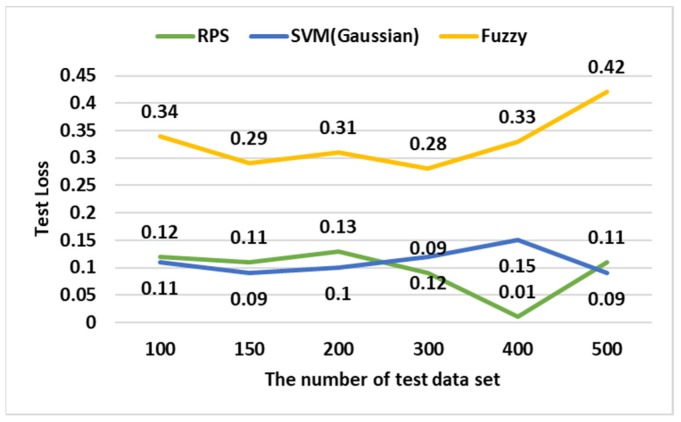
The accuracy according to the test data.

Figure 15 shows a test data loss and over-fitting when RPS, SVM, and fuzzy were used to diagnose parts of a vehicle. The RPS had a loss similar to the SVM and about 0.2 less than the fuzzy. However, since the SVM had over-fitting, RPS was most suitable for part diagnosis of vehicles.

## 5. Conclusions

The LAVS for autonomous vehicle self-diagnosis proposed in this paper consists of the MIGM for communication not only between in-vehicle protocols but also between diagnostic results and the server and the In-VDM for part self-diagnosis and total vehicle self-diagnosis. Here, the In-VDM consists of the RPS for part diagnosis and the NNVS for total vehicle diagnosis. The LAVS guarantees the compatibility of in-vehicle protocols by using the MIGM and the self-diagnosis of a vehicle by using the In-VDM.

The conversion time of the MIGM was improved more than that of the existing in-vehicle gateway by about an average of 28.67%, the error rate of the MIGM was lower than that of the existing in-vehicle gateway by about 0.5%, the computation time of the NNVP was improved by 44.894% and 62.719% more than that of the MPL and the LSTM separately, and the accuracy of the NNVP was higher by about 1% than that of the MLP but similar to that of the LSTM on average. The RPS had a test loss similar to the SVM and about 0.2 less than the fuzzy and the SVM had over-fitting. Therefore, the LAVS was most suitable for not only in-vehicle communication but also part diagnosis and total diagnosis of vehicles.

In addition, this paper would contribute to the following. First, the safety problem will be a major obstacle to supply autonomous vehicles. If the self-diagnosis of autonomous vehicles solves this problem, it will greatly contribute to the supply of autonomous vehicles by changing the perception of customers. Second, an autonomous vehicle executes its self-diagnosis independently, not dependent on the server, so the processing speed will be improved highly. Therefore, it will prevent the accident in advance and in real time.

This study was conducted with government support, and the current experiment was conducted with existing actual data. Further research will be conducted with actual data in real time vehicles and commercialized through industrial and university cooperation.

## Figures and Tables

**Table 1:** Example of the data collection table.

Time	Attribute	Data (or Payload)
2018.11.09 17:00:25:012	Engine voltage	12 V
2018.11.09 17:00:25:054	Tire Pressure	30 psi
2018.11.09 17:00:25:021	Tire temp	50 °C
2018.11.09 17:00:25:008	Front light	9.254 lx
…	…	…
2018.11.09 17:00:26:078	Diagnosis result	75%

**Table 2:** Example of the address table (MOST to CAN).

Attribute	Value
The Number of Divided Messages	n
Current Message Number	1
Source Address	1002
Source Protocol Bus Number	1
Destination Address	3315
Destination Protocol Bus Number	2
Message Priority	1
The 1^st^ message ID	30
….	…
The n^th^ message ID	90

**Table 3:** The results that the random-forest part-diagnosis sub-module (RPS) delivered to the neural network vehicle-diagnosis sub-module (NNVS).

Parts	Engine	Light	Steering	Transmission	…	Break
**RPS result**	–0.251	0.992	0.687	–0.451	…	0.876
